# Dr. John Richards

**Published:** 1913-07

**Authors:** 


Dr. John Richards, Eclectic of Pennsylvania, 1865, a resident of Marion, Wayne County, N. Y., died at Palmyra, May 21, aged 80.

